# Elucidation of impact of heavy metal pollution on soil bacterial growth and extracellular polymeric substances flexibility

**DOI:** 10.1007/s13205-016-0475-x

**Published:** 2016-08-17

**Authors:** Muniswamy David, Paidi Murali Krishna, Jeybalan Sangeetha

**Affiliations:** 1 Environmental Toxicology and Molecular Biology Laboratory, Department of Zoology, Karnatak University, Dharwad, Karnataka 580003 India; 2 Department of Environmental Science, Central University of Kerala, Kasaragod, Kerala 671316 India

**Keywords:** Arsenic, Cadmium, Bioremediation, Extracellular polymeric substances, Polyhydroxybutyric acid (PHBA), FT-IR, FT-Raman

## Abstract

Metal bioaccessibility is an alarming issue in croplands of mining sites due to overloading of toxic metals. Hence, the present study is aimed to determine the overloading of toxic metal in croplands across the Tawag village, Hutti, Raichur, India. Correspondingly, to identify the soil bacterial growth, physiological oxidative stress enzyme activity and surface macromolecular functional group evolution were analysed in and around the toxic metal contaminated sites through FT-IR and FT-Raman spectrometry. The evaluated results attribute that the study area is heavily polluted with the toxic metals such as arsenic, cadmium, chromium, lead and zinc. However, biochemical and 16S rRNA gene sequence homology tree confirmed that the arsenic and cadmium-resistant isolate belongs to *Bacillus* sp. MDPMK-02 and retrieved unique Gene Bank ID KT596811 (accession number) at National Centre for Biotechnology information (NCBI), India. Additionally, sodium arsenite-amended culture media possessing reduced biomass and enhanced the activity of oxidative stress defence enzymes such as superoxide dismutase (SOD) and catalase (CAT) than cadmium chloride-amended medium and control. Subsequently, the infrared (IR) and Raman spectral analytical assessment distinguish that arsenic-treated Gram-positive isolate membrane fetched high percentage of hydration, elevation of surface polysaccharides, proteins and polyhydroxybutyric acid (PHBA) molecular specific stretch intensity compared to cadmium exposures. From these results, the study concluded that the mining wastes significantly pollute the surrounding croplands, and also *Bacillus* sp. MDPMK-02 possesses good chemosensing for cross-protection and bio-adaptation of toxic metal ions. Hence, these isolates can be compiled and implemented in environmental hazardous management techniques such as bioremediation, bioleaching and biodegradation.

## Introduction

Nowadays accumulation of toxic metals through dumping of ore tailings and leachate overflow in and around mining environment is raising the alarm on ecological stress. The mining activities, especially in open-pit mining, typically generate the highest quantities of solid wastes in the form of waste rock and tailings (Mingliang and Haixia 2009). The discharge of acidic mine drainage (AMD) with elevated levels of heavy metals can contaminate the downstream water, agricultural soils, food crops, biota and pose a health risk to residents near the mining areas (Duruibe et al. 2007; Tian et al. 2009; Plumlee and Morman 2011). Heavy metal contamination by mining is a major environmental concern on a global scale, particularly in developing countries (Zhuang et al. 2014). Moreover, cadmium is relatively a typical element not found in the pure state in nature. Its principal uses include protective plating for steel, stabilizers for polyvinyl chloride pigments in plastics and glasses, electrode material in nickel–cadmium batteries and as a component of various alloys and ceramics (Govil et al. 2008). Mine drainage water, waste water from the processing of ore and rainwater run-off from the general ore tailing area and improper disposal of trace elements from industrial wastes ultimately leads to toxic metal especially cadmium pollution (Arhin et al. 2015).

Arsenic (As) is another trace element released from the mine wastes into ground and surface water systems, as well as the geological environment due to their solubility and mobility (Bempah et al. 2013). Arsenic is a silver-gray or white metallic solid element found in nature. The high positive contributions of the anthropogenic mining activities are the major source of the various toxic element, such as As, Pb, and Zn (Marmolejo-Rodríguez et al. 2011). Cropland soil adjacent to an area of historical mining, ore processing and smelting activities reflects the historical background and a mixing of recent toxic heavy metal contamination (Duruibe et al. 2007). Direct atmospheric deposition is occurring near agricultural land from mining and smelting processes and dust particles originating from open tailings ponds (Chrastný et al. 2012).

The soil is heterogeneous in nature, containing many microhabitats that are suitable for microbial growth. Soil microorganisms are playing a vital role in maintaining the balance of soil biogeochemical cycle and soil fertility. These are also involved in various environmentally beneficial applications including bioremediation, biodegradation, biofuel production and bioleaching of mining ore tailings and waste water treatment. Anthropogenic activities, such as rural and industrial development, agricultural pesticides and ore tailings release the toxic heavy metals into soil (Govil et al. 2008). Increasing mining activities has augmented the incidence of percolation of toxic metal ions to the surrounding ecosystem and reached an alarming level (Kulshrestha et al. 2013). The random dumping of hazardous waste in the industrial area could be the main cause of the groundwater and soil contamination (Bhagure and Mirgane 2011). Toxic metallic pollution potentially affects soil microbial diversity by inhibiting physiological mechanisms like respiration, transport of Mg^2+^, sodium–potassium ionic and also reduces the growth (Vijayalakshmi et al. 2011). It is mysterious how the microbial diversity can potentially influence the ecological imbalance in an ecosystem. In order to understand the behaviour of microbial cells at interfaces, it is relevant to study the structure, chemical composition and physicochemical properties of their surfaces.

The toxicity of heavy metal ions possessing strong binding affinity to metal-sensitive functional groups on cell surfaces such as amides, thiol or histidyl moieties even at low concentrations, which leads to conformational modification, denaturation and inactivation or hyperactivity of enzymes, irregular molecular intracellular distribution and organelle integrity (Bae and Chen 2004). Prokaryotes are less compartmentalized compared to eukaryotes, does not have intracellular membrane-bound organelles but chemical composition and structures of the cell wall and cytoplasm are highly complex. They possess diverse molecular and supramolecular structures outside plasma membrane (Naumann 2000). Bacteria are highly sensitive to surrounding environmental conditions even at small changes deny growth and developing endospores. The sustainability of bacterial strains to cope up with sudden changes in the surrounding environment ensures their ecological dominance under stress conditions.

Presently, the major challenge is to study the microbial membrane heterogeneity under toxic stress conditions. The wide microbial surface complexity evolution is further emphasizing the need of single cell membrane architecture analysing techniques. Extra polymeric substances (EPS) are the biopolymer which consists of polysaccharides, proteins, nucleic acids and lipids and plays an important role during biofilm formation, maturation and external protection from hazardous substances (Wang et al. 2012). Electron microscopy and X-ray photoelectron spectroscopy have been used to observe the EPS since past years (Guerrero-Ferreira and Wright 2015). But this technique requires complicated sample preparation, maintains the sample under high vacuum and time consuming, which leads to sample distortion. Other microscopic techniques such as light microscopy, fluorescence microscopy, confocal laser scanning and AFM microscopy have been used for cell intracellular molecular localizations and surface topography (Zemer 2009), but these resolutions remain limited for detailed observation of extra polymeric substances functional analysis.

Apart from the newly evaluated analytical spectral analyses provide accurate and molecular quantitative functional specific intensity for whole bacterial samples. Raman spectroscopy provides advantages over FT-IR spectroscopy because in the former sample need not be dried. Therefore, Raman spectroscopy has been extensively used in the biological sample analysis (Harz et al. 2009). Raman spectroscopy facilitates the analysis of hydrated biofilm cell samples (Movasaghi et al. 2008; Ivleva et al. 2010). Fourier transform infrared (FT-IR) and Raman spectroscopy provide fast and accurate detection of microorganisms. Both these spectral analysis can provide whole organism fingerprint and provide a broad range of biochemical properties about bacteria in a single spectrum (Lu et al. 2011b). FT-IR spectroscopy can characterize bacteria as biochemical information about cellular components including proteins and peptides, carbohydrate, nucleic acids, phospholipids and murein are detectable (Al-Qadiri et al. 2008).

Hence, the present study probes to determine frequent heavy metal pollution and its impact on geochemistry of Tawag crop lands soil, arsenic and cadmium toxic stress sustainable indigenous bacterial defence enzymes activity with respective growth rate. Correspondingly, the toxic metal bio-absorbing bacterial chemosensing extracellular polymeric substances functional group flexibility was analysed through FT-IR and FT-Raman spectrometer.

## Materials and methods

### Collection of soil samples and pre-treatment

Present study was carried out on the croplands of Tawag Village, Hutti gold mining site, Raichur, Karnataka, India (latitude 16°12′17.5″N; longitude 76°38′45.5″E). About five soil samples were collected in a fine plastic polyethene bags and immediately preserved under laboratory conditions. The collected samples were equally mixed together to get composite soil sample for further study. Meanwhile, for metal analysis each sample was grounded and sieved at 0.2 mm plastic mesh.

### Heavy metal quantification

The collected soil samples were subjected to acid (HNO_3_:HCl) digestion followed by filtration (Tijjani et al. 2013) for quantitative determination of their toxic metal content. Resulted filtrate was used as the analyte throughout the experiment for toxic metal quantification by EPA standard method AAS 3050B (USEPA 1996).

### Isolation and identification of heavy metal-resistant bacteria

Five grams of soil composite was suspended into 100 ml liquid minimal salts media (Na_2_HPO_4_-6.78 g, KH_2_PO_4_-5 g, NH_4_Cl-1 g, NaCl-0.5 g and distilled water 1000 ml) in 250-ml conical flasks amended with 25 ppm sodium arsenite (NaAsO_2_) and cadmium chloride (CdCl_2_), then allowed for incubation for 3 days at 30 °C in a shaker incubator at 120 rpm. One millilitre of the turbid liquid medium was diluted up to 10^−6^ with sterile distilled water and 100 µl of 10^−6^ dilute cell suspension was spread on minimal salts agar plates amended with 50 ppm, of toxic metals NaAsO_2_ and CdCl_2_. After 7 days incubation at 37 °C the appeared colonies were screened, morphologically based on its size, shape, colour and optical property. Simultaneously, the Gram’s staining and biochemical tests such as indole, methyl red, Voges–Proskauer, citrate utilization, urease, nitrate reduction, triple sugar iron, gelatinase, starch hydrolysis, catalase, oxidase and H_2_S production test by standard methods (Prescott et al. 2005; Balows et al. 1991).

### Isolate 16S rRNA gene sequence and phylogeny

Microbial DNA extraction was carried out by bacterial genomic DNA isolation kit (KT28A, GeNei™ India) following the manufacturer instructions. The extracted DNA 16S rRNA specific gene was amplified using 400 ng of forward primer F27 5′-AGAGTTTGATCMTGGCTCAG-3′ and reverse primer R1492 5′-TACGGYTACCTTGTTACGACTT-3′ (Boye et al. 1999). The amplification was performed in 100 μl reaction mixture containing 1 μl of DNA, 4 μl of dNTPs (2.5 mM each NT), 10 μl of 10× Taq DNA polymerase assay buffer, 1 μl of Taq DNA polymerase enzyme (3 U/μl) and water. PCR was carried out with initial denaturation 5 min at 94 °C, followed by 35 cycles at 94 °C for 30 s, 30 s at 55 °C and 1 min at 72 °C and a final extension at 72 °C for 5 min. The resulted amplified PCR products were obtained by gel elution extraction spin-50 kit from GeNei, India. The obtained DNA fragments were sequenced by automated dye-terminator method through ABI 3500xL Genetic Analyzer (Bangalore, India). The evaluated 16S rRNA sequence was aligned by ClustalW, evolutionary pairwise distances computed and phylogenic tree was constructed by the neighbour-joining method through MEGA version 6.0 (Tamura et al. 2007). Correspondingly, the resulted gene sequence was submitted to National Centre for Biotechnology information (NCBI) for retrieving a unique gene bank reference ID (accession number).

### Arsenic and cadmium impact on bacterial growth

Prior to determining the arsenic and cadmium impact on bacterial viability, a loop full of pre-culture metal-resistant bacterial inoculums was uniformly spread on MSM agar plates and suspended into 250-ml conical flasks containing 50 ppm of sodium arsenite (NaAsO_2_), 50 ppm of cadmium chloride (CdCl_2_) amended MS liquid media. For every 24-h intermission the CFU and OD at 600 nm were recorded. A typical graph was constructed in between CFU, incubation time and optical density (Kalantari and Ghaffari 2008).

### Arsenic and cadmium inducing ecological stress

In order to illustrate the heavy metal soil pollution inducing ecological stress on indigenous microbes was examined by oxidative stress stabilizing enzymes such as superoxide dismutase (SOD) and catalase (CAT) activity.

#### Extraction of protein for enzymatic assays

About 10 ml pre-culture of bacterial isolate of late log phase was harvested at 4 °C at 3000 rpm for 10 min. The resulted biomass was washed with phosphate buffer saline (PBS) (pH 7.0) and re-centrifugation was done and the pellet was resuspended in the PBS buffer. The individual cells were disrupted by ultrasonication for 30 s burst cycle for a total of 5 min with 1 min cooling period at 4 °C. Simultaneously, cellular debris was removed by centrifugation at 12,000 rpm for 15 min. The supernatants were dialyzed against potassium phosphate buffer and used for enzymatic assay. The protein concentration was determined by the Bradford method (Lenartova et al. 1998; Bradford 1976).

#### Determination of superoxide dismutase (SOD) activity

SOD (EC 1.15.1.1) activities were determined by measuring the photochemical reduction ability of nitro blue toluene (NBT) according to Dhindsa et al. (1981). The test tubes (1.5 ml buffer, 0.2 ml of methionine, 0.1 ml extract with equal amount of NaCO_3_, NBT, riboflavin, EDTA and distill water) were treated for fluorescent illumination at 25 °C in laminar air flow cabinet. After illumination the absorbance was measured at 560 nm. The SOD activity denotes in the units, i.e. the amount of enzyme that inhibits the NBT photoreduction by 50 %, and the enzyme was quantified on the basis of the inhibition percent (Pandey et al. 2013).

#### Determination of catalase (CAT) activity

The CAT activity was determined by measuring the rate of decrease in absorbance at 240 nm of 12.5 mM H_2_O_2_ solution in 50 mM K-phosphate (pH 7.0) at 30 °C. The amount of enzyme per assay was adjusted so that the rate of reaction was kept linear for 2 min. One unit is defined as the amount of enzyme catalysing the decomposition of 1 µM of H_2_O_2_ per minute calculated from the extinction coefficient of H_2_O_2_ at 240 nm of 0.036 cm^2^ µmol^−1^ (Lûck 1963).

### Surface spectral characterization of the isolates

Pre-cultured arsenic and cadmium-resistant isolates biomass (5 mg) was harvested by centrifuging at 3000 rpm and washed with distilled water and centrifuged again at 3000 rpm for 5 min, the resulted pellet was subjected to further spectral analysis.

#### Fourier transform infrared spectrometer (FT-IR)

Prior to inferring the microbial surface molecular biochemistry under heavy metal stress conditions, the harvested biomass (2 mg) pellet was lyophilized in liquid nitrogen tank at −120 °C and vacuum dried at room temperature. About 20 mg of the frozen pellet was finely ground and encapsulated in 200 mg of KBr (Sigma) in order to prepare translucent sample disks with homogenized KBr pellet. The spectra of the lyophilized samples were analysed through Nicolet FT-IR 6700 (Thermo scientific, Dharwad, India) at the mid-IR region, single beamsplitter, DTGS-KBr detector at spectrometer range 4000–400 cm^−1^ of ETC source. All spectra were acquired in turbo mode, by the KBr disc method to get the whole cell surface information specific to the functional groups and the resulted spectra vibration peaks were compared with standard QC libraries of OMNIC (Schuster 1999; Azam et al. 2015).

#### Fourier transform-Raman instrumentation

The EPS molecular functional flexibility is quantitatively determined by Raman spectra. 2 mg biomass of the harvested isolate was resuspended into distilled water in a fine Eppendorf tube (2 ml) and used for further Raman analysis. A wavelength of 400 nm with a laser power of 2 mW was focused onto the samples. The laser beam was focused on EPS extract through a 20× objective. FT-Raman scattering spectra were detected by a charge coupled device (CCD) array detector. The size of each pixel was 16 × 16 µm (Nicolet FT-Raman 6700, Thermo scientific, Dharwad, India). The Raman spectrum was performed with a spectral range of 100–4000 cm^−1^ for all the samples. For measurement at a single location, each full spectral measurement was for a 3-s integration time with 15 spectral accumulations (Huang et al. 2010; Smith and Dent 2005).

### Statistical analysis

Soil geochemical profile results and the level of toxic significance of As and Cd on indigenous bacterial viability (CFU, OD) were analysed statistically using ANOVA multiple comparison tests (post hoc test) in Tukey’s by using SPSS version 16.0 (*P* < 0.05).

## Results and discussion

### Soil quality assessment

Soil is an ultimate ecosystem for aerobic microbes, because it is a reservoir of essential minerals and nutrients and also balances the geochemical composition by cycling phenomenon. In fact, microbes are at the first line of environmental toxicant exposures and bearing resistance. The present assessment noticed that the toxic metals As, Cd, Pb and Zn loading were presented in Table 1. Sample 1, having almost closer to the arsenic, copper and lead permissible limits, where zinc is elevated at 402.63 mg/kg. Subsequently, the soil sample 4 possesses higher amount of toxic metal as compared standard permissible limits of World Health Organization (WHO).

**Table 1:** Toxic heavy metal content across the crop lands of Tawag village (mg/kg)

Soil sample	Mean ± standard deviation (*n* = 3)				
	As	Cd	Cu	Pb	Zn
1^a^	19.43 ± 0.032	1.54 ± 0.02	95.72 ± 0.01	72.42 ± 0.01	402.63 ± 0.015
2	13.017 ± 0.04	ND	61.87 ± 0.02	22.34 ± 0.26	506.24 ± 0.01
3	ND	6.05 ± 0.02	116.76 ± 0.05	82.68 ± 0.28	610.73 ± 0.02
4^b^	26.95 ± 0.032	5.56 ± 0.038	122.91 ± 0.05	72.86 ± 0.04	557.55 ± 1.23
5	11.44 ± 0.18	ND	62.66 ± 0.2	24.11 ± 1.1	56.10 ± 0.05
St. limit.	20		100	100	300

^a^The soil fetching toxic metal content almost nearer to permissible limits

^b^Points out that the soil sample fetching more than permissible limits

Hence, the present assessment progresses about the impact of Hutti goldmine wastes discharge on surrounding croplands of Tawag village and overloading toxic metal status. Present study results attribute that the crop lands of Tawag village possessing As, Cd, Cu, Pb and Zn metals elevation, either by water streams from Hutti gold mine ore tailings during rainfall, leachate overflow and direct usage of ore tailings as a fertilizer composite mixture preparation due to high mineral source. The mining activities enhancing toxic metal weathering in croplands and actively enter into the food web (Zhuang et al. 2014). The historic gold mining site surrounding water bodies and croplands is frequently contaminated with arsenic as determined previously in Mangalore greenstone belt of Northeastern Karnataka, India (Chakraborti et al. 2013). In the present study, quantitative determination of heavy metals enlighten the goldmine ore as major resources for various toxic metals and simultaneously supported by the current hypothesis, viz. contamination of toxic heavy metals overloading in the Tawag village croplands.

### Toxic metal-resistant soil bacteria characterization and identification

**Fig. 1:**
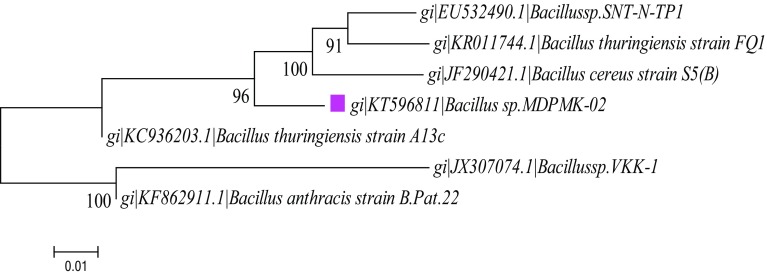
Phylogenies of arsenic and cadmium-resistant isolate bacteria *Bacillus* sp strain MDPMK-02. The *pink* *colour* highlights homology percentage of isolate 16S rRNA sequence with neighbour species

Physiochemical properties of bacterial isolates from toxic metal loading soil territory are presented in Table 2. Bacterial colonies were morphologically irregular, flat in texture, umbonate elevation, Gram positive and rod-shaped polar flagellated cells. The biochemical tests results revealed the production of detectable enzymes catalase, cytochrome oxidase, urease, gelatinase, starch hydrolase and citrate utilization tests results were ubiquitous for the isolate MDPMK-02. These biochemical tests results were compared with Bergey’s Manual of Determinative Bacteriology to confirm that these isolates belong to the genus *Bacillus* sp. The partial 16S rRNA gene sequences of isolate MDPMK-02 exhibits 96 % homology with *Bacillus cereus* strain S5(B) and bearing 0.01 evolutionary distance (Fig. 1). Correspondingly, the NCBI generate the unique Gene Bank accession number as KT596811. Moreover, the morphological, biochemical and molecular assessments results were compared with Bergey’s Manual of Systematic Biotechnology and finally the isolated bacteria were identified as *Bacillus cereus* strain MDPMK-02.

**Table 2:** Characteristics of heavy metal polluted soil territory soil bacteria

Parameters	Colonies
Size	Large
Colony shape	Irregular
Elevation	Umbonate
Margin	Undulate (wavy)
Colony texture	Flat
Motility	Polar flagellated
Optical property	Non-translucent
Colour	Cream
Gram staining	+ve purple, rod shape
Indole	−
Catalase	+
Cytochrome oxidase	+
Urease	+
Casein hydrolase	−
Methyl red	+
Gelatinase	+
H_2_S production	−
Voges–Proskauer	+
Starch hydrolysis	+
Coagulase test	−
Citrate utilization test	+

“+” test positive results; “−” test negative results

### As and Cd toxic potential on bacterial growth

**Fig. 2:**
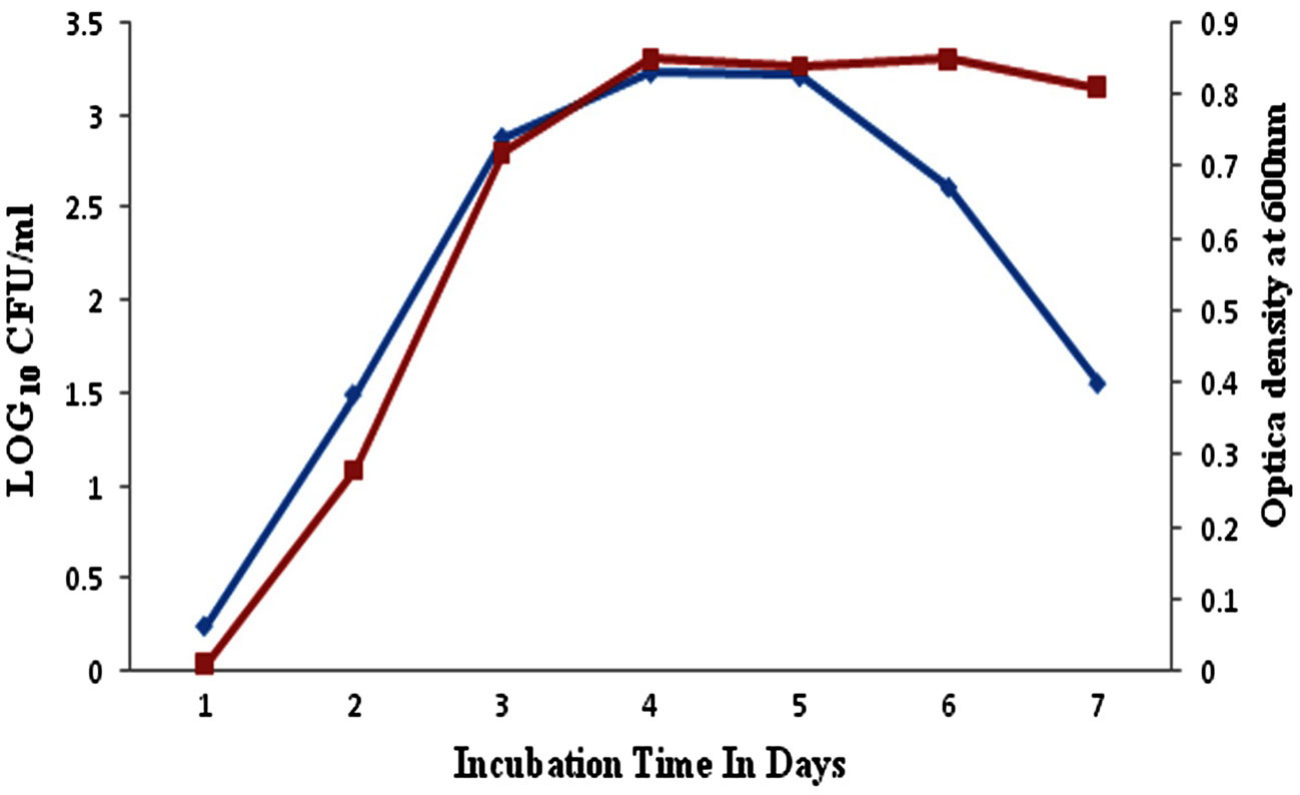
Isolate *Bacillus* sp strain MDPMK-02 viability in presence of arsenic (100 ppm). *Green curve* represents the CFU value and *red curve* indicates optical density during 7 days incubation

**Fig. 3:**
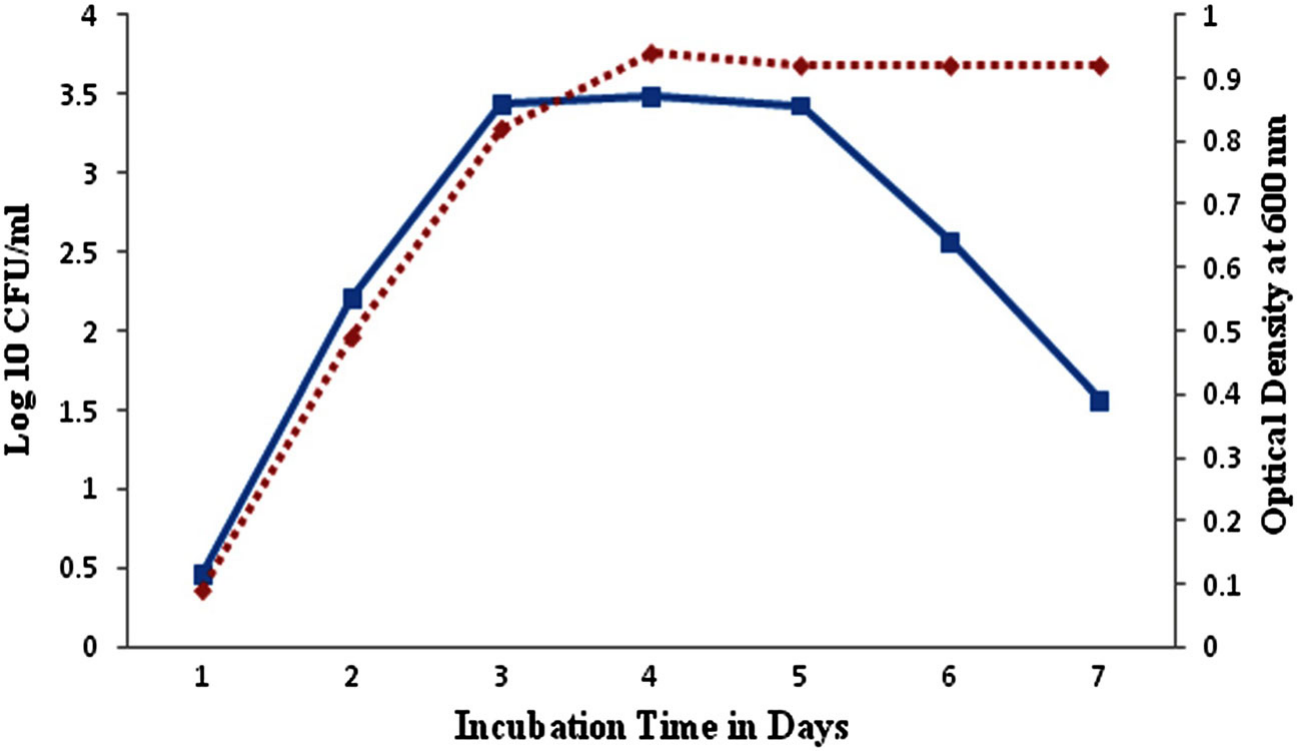
Isolate *Bacillus* sp strain MDPMK-02 viability in presence of cadmium (100 ppm). *Green curve* represents the CFU value and *red curve* indicates optical density during 7 days incubation

Microbial growth impairment was observed successfully by exposing the isolate to toxic metal substances CdCl_2_, NaAsO_2_ individually at 100 ppm for 7 days. The evaluated results illustrate that the arsenic metal-amended liquid and agar media show bell-shaped growth rate and lower CFU than cadmium chloride-amended and control medium. Figure 2 illustrates the isolate MDPMK-02 bell-shaped growth curve contain lag, log and decline phases in the presence of arsenic, while the cadmium-exposed isolate growth curve has clear log, lag, saturation and decline phases which are almost similar to control (Fig. 3).

Simultaneously, arsenic and cadmium impact on bacterial viability observation point out that growth rate of isolate and pattern of normal growth curves. However, the control and cadmium chloride-amended culture media possess equal CFU and OD and show S-shaped growth curve with lag, log, saturation and decline phases, while arsenic amendments disturb the isolate growth rate and they almost lose the saturation/stationary phase in the growth curve, becoming bell shaped. It might be due to early cell death. Previously, Kalantari and Ghaffari (2008) reported that the toxicity of heavy metals such as arsenic, cadmium, iron, chromium amendment impairs the *E. coli* and *B. cereus* growth rates. These reports further supported the evaluation of cadmium and arsenic pollution impact on soil microbes.

### Toxic metal induces physiological stress

**Fig. 4:**
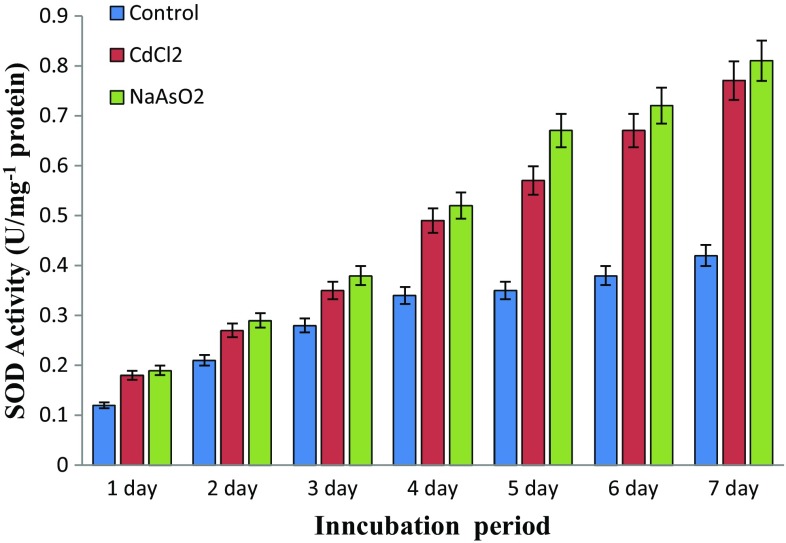
Isolate *Bacillus* sp. superoxide dismutase (SOD) activity under toxic metal-amended culture media supplements (*P* < 0.05)

**Fig. 5:**
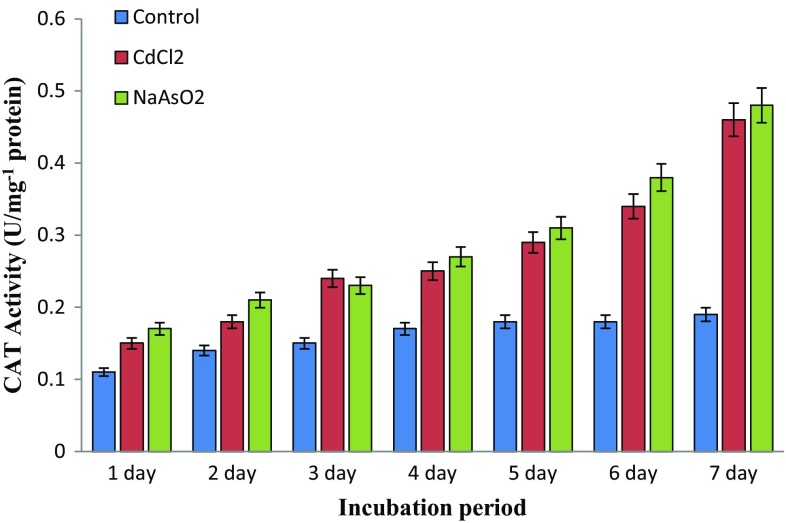
Isolate *Bacillus* sp. catalase (CAT) activity under toxic metal-amended culture media supplement (*P* < 0.05)

Heavy metal polluted soil territory bacteria suffering from physiological stress lead to the production of reactive oxygen species (ROS) such as O_2_^−^, H_2_O_2_ and OH^−^ quantitatively measured by SOD and CAT activity. The present experimental results distinguish both arsenic and cadmium metal contamination inducing physiological stress on native soil microbes. However, Fig. 4 shows the significant elevation of dismutation of superoxide catalysing physiological defence SOD activity level at *P* < 0.05 compared to control, while Fig. 5 demonstrates the peroxide neutralizing catalase activity under metallic stress conditions. These are the milestones for free radical catalysis in physiological defence system and enhancing the sustainability of isolate under metallic ecological stress environments.

Cadmium has the ability to induce physiological stress and altering the protein expression in bacterial cells, in particular, plays an active role in determining the amount of growth factors and other protein (Vido et al. 2001). Cadmium increases oxidative stresses of *S. cerevisiae* which are deficient in antioxidant defence enzymes showing high sensitivity to cadmium (Muthukumar and Nachiappan 2010). Formerly, Banjerdkij et al. (2005) found that the hazardous metal substances may also cause variations in microbial extracellular substances and gene expression leading to morphological, physiological changes, antibiotic resistance and stress tolerance. However, the current experimental task distinguishes the sodium arsenite and cadmium chloride supplemented media reducing the *Bacillus* sp strain MDPMK-02 growth and disturbance in the normal growth curve. Similar observations were made during transitional elemental supplementation reducing the viability of *B. subtilis*, *C. rubrum* and *Pseudomonas* sp. (Vijayalakshmi et al. 2011; Batool et al. 2014).

Correspondingly, the oxidative stress is responsible for free radical production and neutralizing physiological enzyme activity measurement addressing the isolate *Bacillus* sp strain MDPMK-02 oxidative defence flexibility under toxic metal stress conditions. The elevation of dismutation of superoxide catalysing physiological defence SOD activity was observed in the presence arsenic and cadmium metal substance in the culture medium. This converts the superoxide (O_2_^−^) into oxygen and hydrogen peroxide. The resulted hydrogen peroxide is further catalysed by catalase enzyme into water and oxygen (Pandey et al. 2013). The current study also found that toxic arsenic and cadmium metal exposures inducing oxidative stress on soil microbes and evolution of oxidative stress resistance.

### Surface functional spectral assignment of isolate

Unlike other techniques used in microbiology, the spectral examination is a relatively simple method for studying microbial surface molecular functional changes occurring during environmental stress conditions (Simonescu 2012). The bacterial vibrational spectra provide a wide range of biochemical properties about bacteria in a single spectrum, most importantly characteristics of the cell membrane (Kairyte et al. 2012; Booyens and Thantsha 2014). Interestingly, the toxic metal-resistant single bacteria cell surface molecular IR and Raman spectra enlighten the membrane polarity and metal bio-absorbing macromolecular functional banding flexibility under ecological stress conditions (Lu et al. 2011b). Moreover, both IR vibrational spectra and Raman scattering spectral examination provide same and complementary results (Lin et al. 2007; Lu et al. 2011a). Interestingly, both infrared and Raman spectral assessment of isolated *Bacillus* sp strain MDPMK-02 vibrational assignments distinguish the pattern of surface molecular functional group characteristic evolution with respective to toxic metal exposures.

#### FT-IR vibrational band assignment

**Fig. 6:**
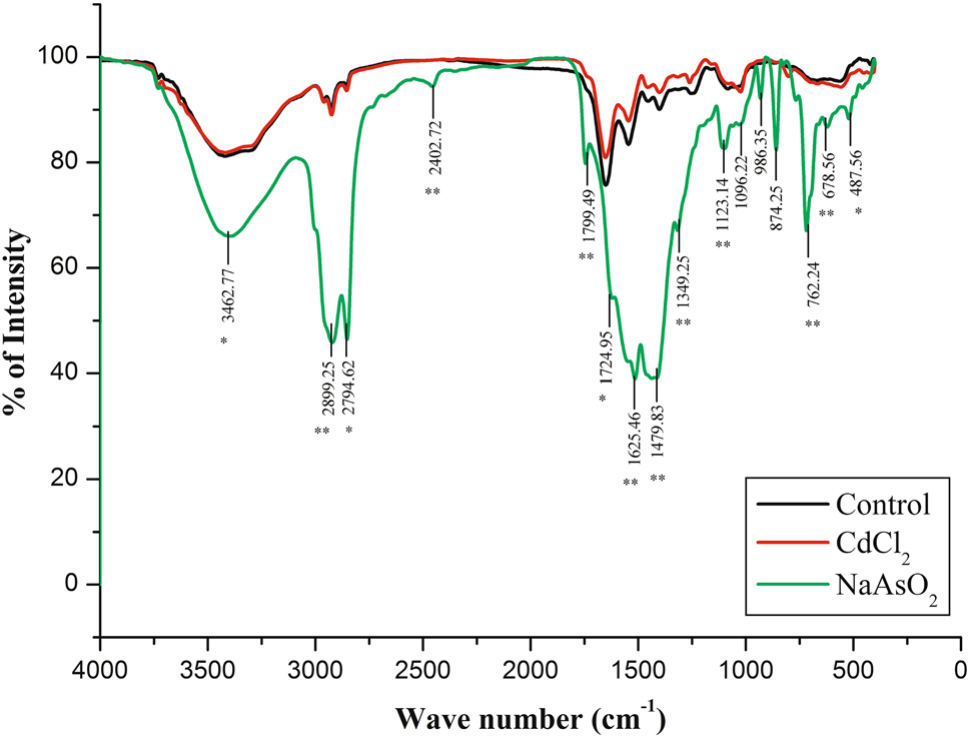
Comparison of FT-IR spectral analysis of *Bacillus* sp. strain MDPMK-02. The *black* and *red* *coloured vibrational lines* indicate the control and cadmium chloride exposed. The *green* *colour* spectra for sodium arsenite (NaAsO_2_) treated conditions. *Asterisk* indicates the common stretching for As, Cd and control cultures, while *double asterisk* points out the evolutionary peaks during sodium arsenite exposures

The infrared spectral inferences of isolated *Bacillus* sp. MDPMK-02 control (black coloured) and exposed to cadmium chloride (in pink) and sodium arsenate (green in colour) are presented in Fig. 6. The vibrational intensity is proportional to the surface cellular molecular functional groups. However, Table 3 represents the major IR absorbance wave regions from 3464.77 cm^−1^ to 3399.14 cm^−1^ (O–H stretching) indicating cell wall hydration, and 2893.25 cm^−1^ 2402.72 cm^−1^ (C–H stretch) for lipids, aliphatic and methyl groups substitution elevation. The region from 1799.49 cm^−1^ to 1724.95 cm^−1^ (C=O, C–N) are hallmarks for membrane surface amide groups synthesis. 1625.46 cm^−1^ (N–H, N=C) is the region for raising the amino radical groups, while from 1498.68 cm^−1^ to 1349.22 cm^−1^ the deep absorption region revealed (C–O–P, C=C, C–H) abundance of surface polysaccharide bounded phosphate moieties, and the region from 1123.14 cm^−1^ to 1096.22 cm^−1^ (C–C=O, C–O–P) specifies the membrane saccharide expression. 874.25 cm^−1^ to 855.69 cm^−1^ (CH–O–P) specifies the phosphodiester bands, and the complex absorbance from the region 678.56 cm^−1^ to 487.56 cm^−1^ (S–H, C–S, S–S and S–O) stretch indicates the functional peptides sulphate, disulphate, thiols, and thiocarbonyl group expression on the membrane surface under toxic metal stress.

**Table 3:** Tentative FT-Raman spectral analyses of isolate *Bacillus* sp. strain MDPMK-02 grown in medium contain sodium arsenite, cadmium chloride for 7 days

S. no	Wave number	Vibrational assignment	Functional group or bimolecular interpretation	In response
1	3462.77 cm^−1a^	O–H str. of hydroxyl groups	Hydration of bacterial cell wall	Arsenic, cadmium and control
2	2899.25 cm^−1a^	C–H stretching of C–H≤	Amino acids in protein polymers, fatty acids	Arsenic, cadmium and control
3	2794.62 cm^−1a^	C–H asymmetric stretching of –CH_3_	Aliphatic fatty acids side chain	Arsenic, cadmium and control
4	2402.72 cm^−1b^	C–H stretch	Aliphatic fatty acid side chain	Arsenic only
5	1799.49–1724.95 cm^−1b^	C=O, C–N	Amide groups of membrane surface α or β protein predominance and lipids	Arsenic only
6	1625.46 cm^−1b^	N–H, N=C	Amides I of α helical structure of proteins	Arsenic only
7	1479.83 cm^−1a^	CH_2_, C–O–P, C=C, C–H, COO	Phosphates groups, phospholipids, fatty acids mixtures	As, Cd and control
8	1349.25 cm^−1b^	PO^−2^ band	Phosphate ionic groups	As only
9	1123.14 cm^−1b^	–C–O–C, C–O stretching	Polysaccharide ring specific vibration	As only
10	1096.22 cm^−1a^	–C=O, C–O–P and CH–O band	Membrane polysaccharides and glycophospholipids	As, Cd and control
11	874.25–855.69 cm^−1b^	C–O–C (str) band	Polysaccharide ring specific vibration	As only
12	762.24 cm^−1b^	C–H stretching for >CH_2_	In fatty acids and protein	As only
13	678.56 cm^−1a^	S–H, C–S, S–O	Sulphate, disulphate, thiols, thiocarbonyl	As, Cd and control
14	487.56 cm^−1a^	S–S stretch	Sulfur groups	As, Cd and control

^a^The vibrational stretching under As, Cd and control culturing

^b^The elevation of vibration stretching for sodium arsenite-amended culture

#### FT-Raman scattering and band assignment

**Fig. 7:**
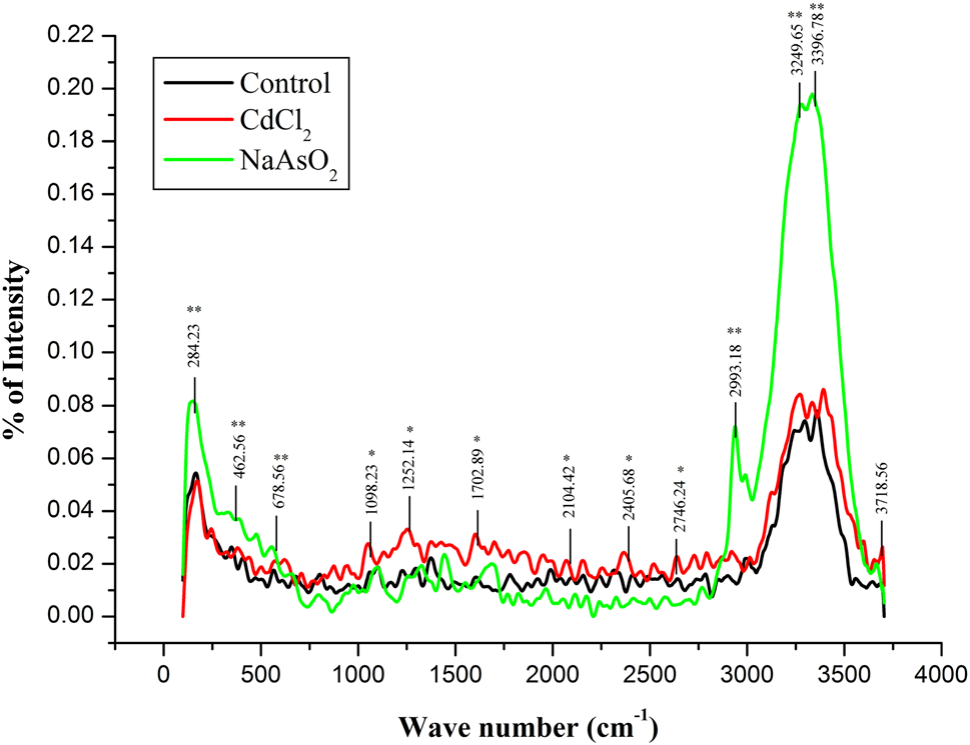
Tentative FT-Raman spectra of *Bacillus* sp. strain MDPMK-02 surface molecular vibrational fingerprint. The *black* and *red* *coloured lines* indicate the control and cadmium chloride exposed. The *green* *colour* spectra for sodium arsenite (NaAsO_2_) treated conditions. *Asterisk* indicates the common stretching for As, Cd and control cultures, while *double asterisk* points out the evolutionary peaks during sodium arsenite exposures

Obviously, the Raman spectral annotation of isolated *Bacillus* sp. strain MDPMK-02 also resumes the surface molecular distribution which is almost similar and complementary to former pointed IR spectroscopy with some exceptions. Figure 7 highlights the cadmium and arsenic resistance isolate bacteria strain showing diverse surface Raman spectral vibrational response under sodium arsenite (in the green curve) and cadmium chloride (in red) individual toxic metal exposures compared to the control (in black curve). From this spectral annotation, we find that the cadmium chloride treatment bacteria possess almost similar molecular functional diversity to the control surface. Simultaneously, Table 4 reveals the Raman light scattering for functional-specific assignment: the elevation scattering regions at 284.23 cm^−1^ (COO^−^) for the carboxylic acid groups of amino acids and the region from 462.56 cm^−1^ to 678.56 cm^−1^ (C–S, S–S) for sulphur bands. The region 1098.23 cm^−1^ (P=O) denotes the membrane phosphodiester bands and in the peak from 2746.24 cm^−1^ to 2993.18 cm^−1^ the C–H stretch indicates the >CH_2_ groups. The major peak regions from 3249.65 cm^−1^ to 3396.78 cm^−1^ (N–H) of amide banding and 3718.56 cm^−1^ of –OH banding are assigned to membrane surface glycopolysaccharide elevation.

**Table 4:** Tentative FT-Raman spectral analyses of isolate *Bacillus* sp strain MDPMK-02

S. no.	Wave number	Vibration assignment	Functional group or bimolecular interpretation	In response
1	284.23 cm^−1^	COO^−^ band stretching	Amino acids	As, Cd and control
2	462.56 cm^−1b^	C–S, S–S disulfide	Sulphide band in protein	As only
3	678.56 cm^−1^	S–H, C–S, S–O	Sulphate, disulphate, thiols, thiocarbonyl	As, Cd and control
4	1098.23 cm^−1a^	P=O symmetric stretching	Phosphodiesters in phospholipids, RNA, DNA	As, Cd and control
5	1252.14 cm^−1a^	C–O–C, C–O	Polysaccharide ring specific	As, Cd and control
6	1702.89–1956.28 cm^−1a^	>C=O starch	For esters in lipid, nucleic acids and carbonic acids	As, Cd and control
7	2104.42 cm^−1a^	C–H stretch	In fatty acids	As, Cd and control
8	2746.24 cm^−1a^	C–H symmetric stretch of –CH_3_	In fatty acids	As, Cd and control
9	2993.18 cm^−1b^	C–H symmetric stretch of >CH_2_	In fatty acids	As, Cd and control
10	3249.65 cm^−1b^	N–H stretch	Amide A in proteins	As only
11	3396.78 cm^−1a^	N–H stretch, N=C str.	Amide A in proteins	As, Cd and control
12	3718.56 cm^−1a^	O–H stretching	Hydration of cell wall	As, Cd and control

^a^The vibrational stretching under As Cd and control culturing

^b^The elevation of vibration stretching in sodium arsenite exposures

However, the IR spectral diagnosis of stress-tolerant biofilm samples, at a mid-IR range of spectrum within 4000–400 cm^−1^ seems to be more optimal than the near-IR range (14,000–4000 cm^−1^) due to an existence of overtone. The major reason is that many biomolecules, such as proteins, lipids, nucleic acids, and carbohydrates have characteristic and specific vibrational fingerprints (Gremlich and Yan 2001; Krafft 2004). The predominant IR bands are hydroxyl groups (3462.77 cm^−1^: O–H stretching), 2899.25 cm^−1^ for C–H stretch, amide I (1724.95–1799.46 cm^−1^ for C=O stretch mode), amide II (1625 cm^−1^ N–H, N=C stretch modes), phosphate groups (1479.83–1123.14 cm^−1^: C–O–P, –C–O stretching), asymmetric or symmetric phosphate (near to 1096 cm^−1^: PO^2−^), polysaccharides (874.25–855.69 cm^−1^: C–O–C banding) and sulphur, thiocarbonyl groups (678.56–487.56 cm^−1^: S–H, C–S, S–O and S–S banding) for arsenic and cadmium metal treated isolate, respectively. Former (Jian-hua et al. 2007; Palaniappan and Vijayasundaram 2008; Saikia 2010; Davis and Mauer 2010) Raman and infrared spectral biological diagnosis and bands stretching assignments results further strengthen the present study results.

Obviously, the isolate surfaces enhance Raman scattering spectrum also prove the band-specific vibrations are similar to former pointed IR spectra. Meanwhile the strong intensity for polar bands at the regions of 284.23 cm^−1^ (S–H, S–S stretches); 2993.18–284.23 cm^−1^ (C–H symmetry of >CH_2_ stretching); 3249.65–3396.78 cm^−1^ (O–H stretching), while for non-polar functional band intensity is weak. The current single bacteria cell surface molecular IR and Raman spectral analytical study enlightens the surface chemical information of Gram-positive bacteria *Bacillus cereus* strain MDPMK-02 under arsenic and cadmium exposures.

## Conclusion

The present geochemical status results attribute that study area Tawag village polluted by toxic metals such as arsenic, cadmium, copper, lead and zinc. Correspondingly, indigenous metal-resistant isolate bacteria *Bacillus* sp. MDPMK-02 possess good growth and physiological stress flexibility. Nevertheless, the IR spectral and FT-Raman surface scattering vibrational spectrum enlighten about the Gram-positive bacteria enhancing the surface polarity for metal bio-absorbance and chemotactic sensing for environmental changes. These results prove that *Bacillus* sp. MDPMK-02 could be considered and used in bioleaching, bioremediation and biodegradation of mining and industrial waste treatment.

## References

[CR1] Al-Qadiri HM, Lin M, Al-Holy MA, Cavinato AG, Rasco BA (2008). Detection of sublethal thermal injury in *Salmonella enterica* serotype Typhimurium and *Listeria monocytogenes* using Fourier transform infrared (FT-IR) spectroscopy (4000 to 600 cm^−1^). J Food Sci.

[CR2] Arhin E, Boansi AO, Zango MS (2015). Trace elements distributions at Datoko-Shega artisanal mining site, northern Ghana. Environ Geochem Health.

[CR3] Azam M, Khan A, Muzzafar D, Faryal R, Siddiqi S, Ahmad R, Chauhdry A, Rehman I (2015). Structural, surface, in vitro bacterial adhesion and biofilm formation analysis of three dental restorative composites. Materials.

[CR4] Bae W, Chen X (2004). Proteomic study for the cellular responses to Cd^2+^ in *Schizosaccharomyces pombe* through amino acid-coded mass tagging and liquid chromatography tandem mass spectrometry. Mol Cell Proteomics.

[CR5] Balows A, Truper HG, Dworkin M, Harder W, Schleifer KH (1991). Prokaryotes: a handbook on the biology of bacteria.

[CR6] Banjerdkij P, Vattanaviboon P, Mongkolsuk S (2005). Exposure to cadmium elevates expression of genes in the OxyR and OhrR regulons and induces cross-resistance to peroxide killing treatment in *Xanthomonas campestris*. Appl Environ Microbiol.

[CR7] Batool R, Yrjälä K, Hasnain S (2014). Impact of environmental stress on biochemical parameters of bacteria reducing chromium. Braz J Microbiol.

[CR8] Bempah CK, Ewusi A, Obiri-Yeboah S, Asabre SB, Mensah F, Boateng J, Voigt HJ (2013). Distribution of arsenic and heavy metals from mine tailings dams at obuasi municipality of Ghana. Am J Eng Res (AJER).

[CR9] Bhagure GR, Mirgane SR (2011). Heavy metal concentrations in groundwaters and soils of Thane Region of Maharashtra, India. Environ Monit Assess.

[CR0011] Booyens J, Thantsha MS (2014). Fourier transform infra-red spectroscopy and flow cytometric assessment of the antibacterial mechanism of action of aqueous extract of garlic (Allium sativum) against selected probiotic Bifidobacterium strains. BMC Complement Altern Med.

[CR10] Boye K, Høgdall E, Borre M (1999). Identification of bacteria using two degenerate 16S rDNA sequencing primers. Microbiol Res.

[CR1000] Bradford MM (1976). A Rapid and Sensitive Method for the Quantitation of Microgram Quantities of Protein Utilizing the Principle of Protein-Dye Binding. Anal Biochem.

[CR11] Chakraborti D, Rahman Mohammad Mahmudur, Murrill M, Das R, Siddayya Patil SG, Sarkar A, Dadapeer HJ, Yendigeri S, Ahmed R, Das KK (2013). Environmental arsenic contamination and its health effects in a historic gold mining area of the magalure greenstone belt of Northeastern Karnataka, India. NIH Public Access.

[CR12] Chrastný V, Vaněk A, Teper L, Cabala J, Procházka J, Pechar L, Drahota P, Penížek V, Komárek M, Novák M (2012). Geochemical position of Pb, Zn and Cd in soils near the Olkusz mine/smelter, South Poland: effects of land use, type of contamination and distance from pollution source. Environ Monit Assess.

[CR13] Davis R, Mauer LJ (2010). Fourier transform infrared (FT–IR) spectroscopy: a rapid tool for detection and analysis of foodborne pathogenic bacteria. Curr Res Technol Educ.

[CR14] Dhindsa RH, Plumb-Dhindsa P, Thorpe TA (1981). Leaf senescence correlated with increased level of membrane permeability, lipid peroxidation and decreased level of SOD and CAT. J Exp Bot.

[CR15] Duruibe JO, Ogwuegbu MOC, Egwurugwu JN (2007). Heavy metal pollution and human biotoxic effects. Int J Phys Sci.

[CR16] Govil PK, Sorlie JE, Murthy NN, Sujatha D, Reddy GLN, Rudolph-Lund K, Krishna AK, Rama Mohan K (2008). Soil contamination of heavy metals in the Katedan Industrial Development Area, Hyderabad, India. Environ Monit Assess.

[CR17] Gremlich HU, Yan B (2001). Infrared and Raman spectroscopy of biological materials.

[CR18] Guerrero-Ferreira RC, Wright ER (2015). Zernike phase contrast cryo-electron tomography of whole bacterial cell. NIH Public Access.

[CR19] Harz M, Rosch P, Popp J (2009). Vibrational spectroscopy—a powerful tool for the rapid identification on microbial cells at the single-cell level. Cytometry A.

[CR20] Huang WE, Li M, Jarvis RM, Goodacre R, Banwart SA (2010). Shining light on the microbial world: the application of Raman microscopy. Adv Appl Microbiol.

[CR21] Ivleva NP, Wagner M, Horn H, Niessner R, Haisch C (2010). Raman microscopy and Surface-Enhanced Raman Scattering (SERS) for in situ analysis of biofilms. J Biophotonics.

[CR22] Jian-hua PAN, Rui-xia LIU, Hong-xiao T (2007). Surface reaction of *Bacillus cereus* biomass and its biosorption for lead and copper ions. J Environ Sci.

[CR23] Kairyte K, Luksiene Z, Pucetaite M, Sablinskas V (2012). Differentiation of bacterial strains by means of surface enhanced FT-Raman spectroscopy. Lith J Phys.

[CR24] Kalantari N, Ghaffari S (2008). Evaluation of toxicity of heavy metals for *Escherichia coli* growth. Iran J Environ Health Sci Eng.

[CR25] Krafft C (2004). Bioanalytical applications of Raman spectroscopy. Anal Bioanal Chem.

[CR26] Kulshrestha S, Awasthi A, Dabral SK (2013). Assessment of heavy metals in the industrial effluents, tube-wells and municipal supplied water of Dehradun, India. J Environ Sci Eng.

[CR500] Lenartova V, Holovska K, Javorski P (1998). The influence of mercury on the ant ioxidant enzyme activity of rumen bacteria Streptococcus bovis and Selenomonas ruminantium. FEMS Microbiol Ecol.

[CR27] Lin SY, Li MJ, Cheng WT (2007). FT-IR and Raman vibrational microspectroscopies used for spectral biodiagnosis of human tissues. Spectroscopy.

[CR28] Lu X, Al-Qadiri HM, Lin M, Rasco BA (2011). Application of Mid-infrared and Raman Spectroscopy to the study of bacteria. Food Bioprocess Technol.

[CR29] Lu X, Rasco B, Jabal JMF, Eric Aston D, Lin M, Konkel ME (2011). Investigating antibacterial effects of garlic (*Allium sativum*) concentrate and garlic-derived organosulfur compounds on *Campylobacter jejuni* by using Fourier transform infrared spectroscopy, Raman spectroscopy, and electron microscopy. Appl Environ Microbiol.

[CR30] Lûck H, Bergmeyer HU (1963). Catalase. Methods of enzymatic analyses.

[CR31] Marmolejo-Rodríguez AJ, Sánchez-Martínez MA, Romero-Guadarrama JA, Sánchez-González A, Magallanes-Ordóñez VR (2011). Migration of As, Hg, Pb, and Zn in arroyo sediments from a semiarid coastal system influenced by the abandoned gold mining district at El Triunfo, Baja California Sur, Mexico. J Environ Monit.

[CR32] Mingliang ZMZ, Haixia WHW (2009). Characteristics on soil heavy metals pollution around mine waste piles. Int Conf Environ Sci Inf Appl Technol.

[CR33] Movasaghi Z, Rehman S, Rehman IU (2008). Fourier transforms infrared (FTIR) spectroscopy of biological tissues. Appl Spectrosc Rev.

[CR34] Muthukumar K, Nachiappan V (2010). Cadmium-induced oxidative stress in *Saccharomyces cerevisiae*. Indian J Biochem Biophys.

[CR35] Naumann D (2000) Infrared spectroscopy in microbiology. In: Encyclopedia of analytical chemistry. Wiley, pp 102–131. doi:10.1002/9780470027318.a0117

[CR36] Palaniappan PR, Vijayasundaram V (2008). FTIR study of arsenic induced biochemical changes on the liver tissues of fresh water fingerlings *Labeo rohita*. Rom J Biophys.

[CR37] Pandey S, Barai PK, Maiti TK (2013). Influence of heavy metals on the activity of antioxidant enzymes in the metal resistant strains of Ochrobactrum. J Environ Biol.

[CR38] Plumlee GS, Morman SA (2011). Mine wastes and human health. Elements.

[CR001] Prescott LM, Harley JP, Klein DA (2005). Microbiology.

[CR39] Saikia BJ (2010). Fourier transform infrared spectroscopic characterization of kaolinite from Assam and Meghalaya, Northeastern India. J Mod Phys.

[CR40] Schuster KC (1999). FTIR spectroscopy applied to bacterial cells as a novel method for monitoring complex biotechnological processes 1. Vib Spectrosc.

[CR41] Simonescu CM (2012) Application of FTIR spectroscopy in environmental studies. In: Advanced aspects of spectroscopy. INTECH, p 36. doi:10.5772/48331

[CR42] Smith E, Dent G (2005). Modern Raman spectroscopy—a practical approach.

[CR43] Tamura K, Dudley J, Nei M, Kumar S (2007). MEGA4: molecular evolutionary genetics analysis (MEGA) software version 4.0. Mol Biol Evol.

[CR44] Tian D, Zhu F, Yan W, Fang X, Xiang W, Deng X, Wang G, Peng C (2009). Heavy metal accumulation by panicled goldenrain tree (*Koelreuteria paniculata*) and common elaeocarpus (*Elaeocarpus decipens*) in abandoned mine soils in southern China. J Environ Sci (China).

[CR45] Tijjani N, Dioha IJ, Alhassan B, Eleri AI, Lawal AM, Muhammad I (2013). Determination of some heavy metals in soil samples obtained from Rimi Local Government in Katsina State, Nigeria. Chem Mater Res.

[CR46] Vido K, Spector D, Lagniel G, Lopez S, Toledano MB, Labarre J (2001). A proteome analysis of the cadmium response in *Saccharomyces cerevisiae*. J Biol Chem.

[CR47] Vijayalakshmi S, Usha V, Riyaz Ahmad R (2011). In vitro responses of various bacteria to cadmium chloride: growth and biochemical analysis. Asian J Exp Biol Sci.

[CR48] Wang LL, Wang LF, Ren XM, Ye XD, Li WW, Yuan SJ, Sun M, Guo-Ping S, Han-Qing YU, Wang XK (2012). pH dependence of structure and surface properties of microbial EPS. Environ Sci Technol.

[CR49] Zemer G (2009). New fluorescence microscopy methods for microbiology: sharper, faster and quantitative. NIH Public Access.

[CR50] Zhuang P, Lu H, Li Z, Zou B, McBride MB (2014). Multiple exposure and effects assessment of heavy metals in the population near mining area in South China. PLoS One.

